# Genome adaptive evolution of *Lactobacillus casei* under long-term antibiotic selection pressures

**DOI:** 10.1186/s12864-017-3710-x

**Published:** 2017-04-24

**Authors:** Jicheng Wang, Xiao Dong, Yuyu Shao, Huiling Guo, Lin Pan, Wenyan Hui, Lai-Yu Kwok, Heping Zhang, Wenyi Zhang

**Affiliations:** 1 Key Laboratory of Dairy Biotechnology and Engineering, Ministry of Education, Inner Mongolia Agricultural University, Hohhot, Inner Mongolia 010018 China; 2 Key Laboratory of Dairy Products Processing, Ministry of Agriculture, Inner Mongolia Agricultural University, Hohhot, 010018 Inner Mongolia China

**Keywords:** *Lactobacillus casei* Zhang, Amoxicillin, Gentamicin, Biosafety

## Abstract

**Background:**

The extensive use of antibiotics in medicine has raised serious concerns about biosafety. However, the effect of antibiotic application on the adaptive evolution of microorganisms, especially to probiotic bacteria, has not been well characterized. Thus, the objective of the current work was to investigate how antibiotic selection forces might drive genome adaptation using *Lactobacillus* (*L*.) *casei* Zhang as a model.

**Methods:**

Two antibiotics, amoxicillin and gentamicin, were consistently applied to the laboratory culture of *L. casei* Zhang. We then monitored the mutations in the bacterial genome and changes in the minimum inhibitory concentrations (MICs) of these two antibiotics along a 2000-generation-cultivation lasted over 10 months.

**Results:**

We found an approximately 4-fold increase in the genome mutation frequency of *L. casei* Zhang, i.e. 3.5 × 10^-9^ per base pair per generation under either amoxicillin or gentamicin stress, when compared with the parallel controls grown without application of any antibiotics. The increase in mutation frequency is significantly lower than that previously reported in *Escherichia* (*E*.) *coli*. The rate of *de novo* mutations, i.e. 20 per genome, remained low and stable throughout the long-term cultivation. Moreover, the accumulation of new mutations stopped shortly after the maximum bacterial fitness (i.e. the antibiotic MICs) was reached.

**Conclusions:**

Our study has shown that the probiotic species, *L. casei* Zhang, has high genome stability even in the presence of long-term antibiotic stresses. However, whether this is a species-specific or universal characteristic for all probiotic bacteria remains to be explored.

**Electronic supplementary material:**

The online version of this article (doi:10.1186/s12864-017-3710-x) contains supplementary material, which is available to authorized users.

## Background

The evolution of antibiotic resistance in bacteria has long been an important issue because of the wide application of antibiotics in medicine [1]. The recently developed next generation sequencing technology has greatly improved the process of DNA sequencing. It has also become an affordable tool for characterizing bacterial genome evolution by monitoring the genome changes along long-term laboratory growth. Such approach, adaptive laboratory evolution (ALE), is used for analyzing the evolutionary phenomena of bacteria in a controlled laboratory setting; and it has revealed new insights into microbial genetic adaptations under certain culture conditions [2]. The first description of ALE experiments can be dated back to the study published by Russel in 1893 [3]. Currently, most studies concerning phenotypic and genotypic changes in adaptive evolution are limited to the species *Escherichia* (*E*.) *coli* [4].

The genus *Lactobacillus* is a major part of the lactic acid bacteria (LAB) group that consists of more than 200 known species and subspecies [5]. Because of its ability to ferment raw materials, *Lactobacillus* has a long history of safe use in food production, such as yogurt, cheese, beer, wine, and other fermented foods. Our research team has previously established a gene catalogue of *Lactobacillus* by comparative genomics analysis. Particularly, our work has described a broad and diverse carbohydrate- and protein-modifying genes, as well as the presence of multiple novel CRISPR-Cas immune systems. Moreover, our data revealed how the *Lactobacillus* host interaction factors and bacteriocins affect their natural and industrial environments, and the mechanisms of these bacteria in withstanding stress during technological processes [6]. Apart from being used as starter bacteria in food fermentation, *Lactobacillus* strains that have known health-promoting effects are also applied as probiotics [7]. These strains are widely used in functional fermented dairy products, veterinary medicines, and feed additives [8, 9].

Recently, there are growing interests in the combined use of probiotics and antibiotics to treat gastrointestinal disorders [10]. One major mechanism that renders previously non-resistant bacteria to become resistant to a particular antibiotic is the selection of mutated subpopulations that carry obliterated antibiotic target genes or have acquired novel abilities to remove or deactivate the effective drug [11]. Thus, frequent exposure to an antibiotic-containing environment may make the probiotics more prone to gain additional antibiotic resistance capacity. These bacteria have to reach and be present at least temporarily in the intestines of humans and animals to exert their beneficial effects. During their transit through the host gastrointestinal tract, the mutated antibiotic resistance genes will be directly exposed to other co-occurring gut microbes [12]. Meanwhile, there is an increased chance of interaction between the probiotic bacteria and the complex colon gut microbial community. Despite the potential risk, the evolutionary adaptation of probiotics towards antibiotic resistance has not been adequately studied. Only one work regarding this topic has been published up to now, which characterized the spontaneous drug resistance of *Lactobacillus* at the biochemical level [13].

Therefore, the objective of the current work was to investigate how antibiotic selection forces might drive the genome adaptation in *Lactobacillus* (*L*.) *casei* Zhang. *Lactobacillus casei* Zhang is a probiotic strain isolated from koumiss. It is highly resistant to acid and bile stresses; and it exhibits anti-bacterial and anti-oxidative properties [14, 15]. One obvious advantage of using *L. casei* Zhang as a model is the availability of the detailed genome sequence information. The genome of *L. casei* Zhang consists of a circular chromosome (2,861,848 base pair, bp) and a single plasmid (36 kilobase, kb) [16–18]. The information serves as a reference to identify any mutations and genomic changes. Our study monitored the genotypic and phenotypic changes of *L. casei* Zhang along a 2000-generation-cultivation lasted over 10 months under three growth conditions with or without antibiotics (amoxicillin or gentamicin). To our knowledge, this is the first report investigating the antibiotic-driven genome adaptation of a common food use LAB.

## Methods

### Bacterial growth conditions

The bacterium, *L. casei* Zhang, was used in the current study. It was grown either in de Mann-Rogosa-Sharpe (MRS) broth (CM0359,OXOID) or LAB susceptibility test medium (LSM) [19]. The LSM was made of 90% Iso-sensitest medium (IST; CM0473, OXOID) and 10% MRS.

### Experimental evolution

The laboratory evolution experiments were designed based on the standard protocol described by Pena-Miller et al. [20]. Briefly, an original stock of *L. casei* Zhang was subcultured twice in MRS broth. Then they were streaked onto MRS agar plates. After cultivating at 37 °C for 72 h, three colonies were randomly picked and were separately inoculated into three tubes of 5 mL LSM supplemented with gentamicin (1 μg/mL) (strain G), amoxicillin (0.5 μg/mL) (strain A), or without any antibiotics (strain C). The antibiotic concentrations used were the half maximum inhibitory concentrations (IC_50_) of *L. casei* Zhang determined previously (unpublished data). Each culture was continuously propagated by transferring 1% (v/v, 50 μL) of the original culture into fresh medium every 24 h. Approximately, 100-fold daily bacterial growth represented nearly 6.6 generations per subculture. Samples were collected for fitness evaluation and identification of *de novo* mutations every 200 generations. Frozen stocks were prepared regularly along the long-term cultivation. Strains were numbered in consecutive order according to their generations.

### Evaluation of fitness as represented by minimum inhibitory concentrations (MICs)

The phenotypic changes of the bacterial cultures were measured by the gentamicin and amoxicillin MICs, which were determined using the broth macrodilution method described by Shao et al. [21]. Briefly, bacterial suspensions with turbidity equivalent to McFarland standard 1 (~3 × 10^8^ cfu/mL) were prepared. They were then diluted by 500 folds (~6 × 10^5^ cfu/mL). Doubling dilutions of antibiotics, ranging from 0.25 to 256 μg/ml for both gentamicin and amoxicillin, were freshly prepared in LSM broth. Each antibiotic-containing or control tube was then inoculated with an equal volume of the respective diluted bacterial suspension (i.e. a final bacterial concentration of 3 × 10^5^ cfu/mL). The MIC endpoints were read after 48 h incubation at 37 °C under strictly anaerobic conditions. The assay was repeated three times.

### Identification of *de novo* mutations

The adapted strains were cultured under anaerobic conditions in LSM broth at 37°C, followed by DNA extraction with a bacterial DNA extraction kit (OMEGA D3350-02) according to the manufacturer’s instructions. Genomic DNA samples were quantified by using a TBS-380 fluorometer (Turner BioSystems Inc., Sunnyvale, CA). The DNA quality was checked by spectrophotometry. Qualified DNA samples (OD_260/280_ = 1.8 ~ 2.0, > 6ug) were utilized to construct libraries of 200 to 300 bp fragments.

We selected and sequenced 50 genomes at generations 200, 400, 600, 800, 1000, 1200, 1400, 1800, and 2000. For all sequencing, at least 3 μg genomic DNA was used for sequencing library construction. Paired-end libraries with an insert size of ~300 bp were prepared following the Illumina’s standard genomic DNA library preparation procedures. Purified genomic DNA was sheared into smaller fragments to a desirable size using Covaris fragmentation, and blunt ends were generated using T4 DNA polymerase. After adding an ‘A’ base to the 3’ end of the phosphorylated DNA fragments, adapters were ligated to the ends. The desired fragments were purified by agarose gel electrophoresis before being selectively enriched and amplified by polymerase chain reaction (PCR). An index tag was introduced via an adapter during PCR. A library quality test was then performed, and the qualified Illumina pair-end library was used for Illumina Hiseq 2000 (Illumina Inc. U.S.A) sequencing. The read lengths were 100 bp. An average of 617.92 Mb of high-quality data were generated for each strain, corresponding to a sequencing depth of 157 to 282 folds.

For variant calling, the raw sequence reads were imported into CLC Bio Genomics Workbench V8.5.1 (CLC Inc., Aarhus, Denmark) and mapped against the *L. casei* Zhang genome with 80% identity and a length fraction setting of 0.5. Two built-in variant detection tools, namely Fixed Ploidy Variant Detection and Low Frequency Variant Detection, were used to call mutations. The parameters of the former detector were single nucleotide variation (SNV) coverage of >20, variant probability of 90%, and ploidy set at 1 (high-quality call set), whilst the parameters of the latter detector were the same level of SNV coverage, minimum frequency of 1%, and significance of 1% (lenient call set). Synonymous or non-synonymous sites were discriminated by using the protocol of Amino Acid Changes available in the Functional Consequences module.

To identify true *de novo* mutations that occurred during the long-term cultivation, the common SNVs shared by multiple strains in the high-quality call set were filtered out, as these might have been natural SNVs pre-existent before the experiment. To track when the *de novo* mutations happened and their stability, the lenient call set was analyzed. For each reliable *de novo* mutation identified, we determined the generations of its first and last presence along the adaptive evolution experiment. Ka/Ks ratio was calculated by dividing the number of non-synonymous substitutions per non-synonymous site (Ka) by the number of synonymous substitutions per synonymous site (Ks) [22]. The *L. casei* codon usage and its frequency were obtained from Kazusa DNA Research Institute (KDRI, http://www.kazusa.or.jp/codon/).

To verify the identified variants, a set of reliable SNVs was randomly selected and sequenced by Sanger method. Sequencing of the amplicons was performed by Shanghai Majorbio Bio-Pharm Technology Co., Ltd.

## Results

### Long-term evolution of *L. casei* Zhang with and without antibiotic exposure

We monitored the evolution of *L. casei* Zhang along a 2000-generation-cultivation under three experimental conditions, i.e. with amoxicillin (strain A) or gentamicin (strain G), and control without any antibiotics (strain C). We assessed the strain fitness by monitoring the changes in the amoxicillin and gentamicin MICs at different time points in each case. At generation 0, the MICs for amoxicillin and gentamicin were the same (2 μg/mL). The amoxicillin MIC for strain A increased to a maximum level (8 μg/mL) after subculturing for 400 generations, while the gentamicin MIC for strain G reached 32 μg/mL after subculturing for 1200 generations (Tables 1 and 2). The MIC of the control, strain C, remained unchanged. This indicates that strains A and G had increased fitness towards amoxicillin and gentamicin respective to the exposed antibiotics. The rate of adaptation to amoxicillin was about three-fold higher than that of gentamicin.

**Table 1:** Changes in gentamicin minimum inhibitory concentration (MIC) of *Lactobacillus casei* Zhang during long-term cultivation in lactic acid bacteria susceptibility test medium (LSM) with gentamicin (μg/mL)

Generation	MIC (Gentamicin μg/mL)		
	G1	G2	G3
0	2	2	2
200	8	8	4
400	8	8	8
600	8	8	8
800	16	16	16
1000	16	16	16
1200	32	32	32
1400	32	32	32
1600	32	32	32
1800	32	32	32
2000	32	32	32

**Table 2:** Changes in amoxicillin minimum inhibitory concentration (MIC) of *Lactobacillus casei* Zhang during long-term cultivation in lactic acid bacteria susceptibility test medium (LSM) with amoxicillin (μg/mL)

Generation	MIC (Amoxicillin μg/mL)		
	A1	A2	A3
0	2	2	2
200	4	4	4
400	4	8	4
600	8	8	8
800	8	8	8
1000	8	8	8
1200	8	8	8
1400	8	8	8
1600	8	8	8
1800	8	8	8
2000	8	8	8

### Accumulation of *de novo* mutations

For every 100 generations, frozen stocks of strains A, G, and C were prepared. They were recovered at the end of the experiment and were sequenced. *De novo* mutations were identified by comparing the obtained sequences with the reference genome of *L. casei* Zhang [17]. Possibly, due to the physical damage exerted to the bacteria during frozen stock preparation, a few of the samples for certain time points (from culture conditions of C3, G2, A1, and A2) could not be recovered or grew poorly after being thawed. We therefore excluded these problematic samples in the genomic analysis. Fortunately, representative samples were obtained to cover all time points along the 10-month experiment, so that a complete image of evolution could be presented.

We randomly selected 11 mutations and performed Sanger sequencing to validate mutation calling. Every SNV-containing locus was sequenced from both directions, covering approximately 400 bp. Nine out of the 11 variants found by Sanger sequencing were validated, which is comparable to a previous study [23]. The complete list of the confirmed SNVs with the respective predicted changes at amino acid level is provided in Additional file 1: Table S1. Such results validated our sequencing and mutation calling methodologies in identifying *de novo* mutations. The mutations identified were distributed to a high proportion of the sequence reads. Since we excluded the common SNPs and identified mutations shared across strains A, G, and C in subsequent analysis, the accumulated *de novo* mutations found in this study truly reflected the genomic changes happened during the long-term propagation. These *de novo* mutations were regarded as fixed variants. We traced these *de novo* mutations in the lenient criteria call set and identified when they first occurred.

**Fig. 1:**
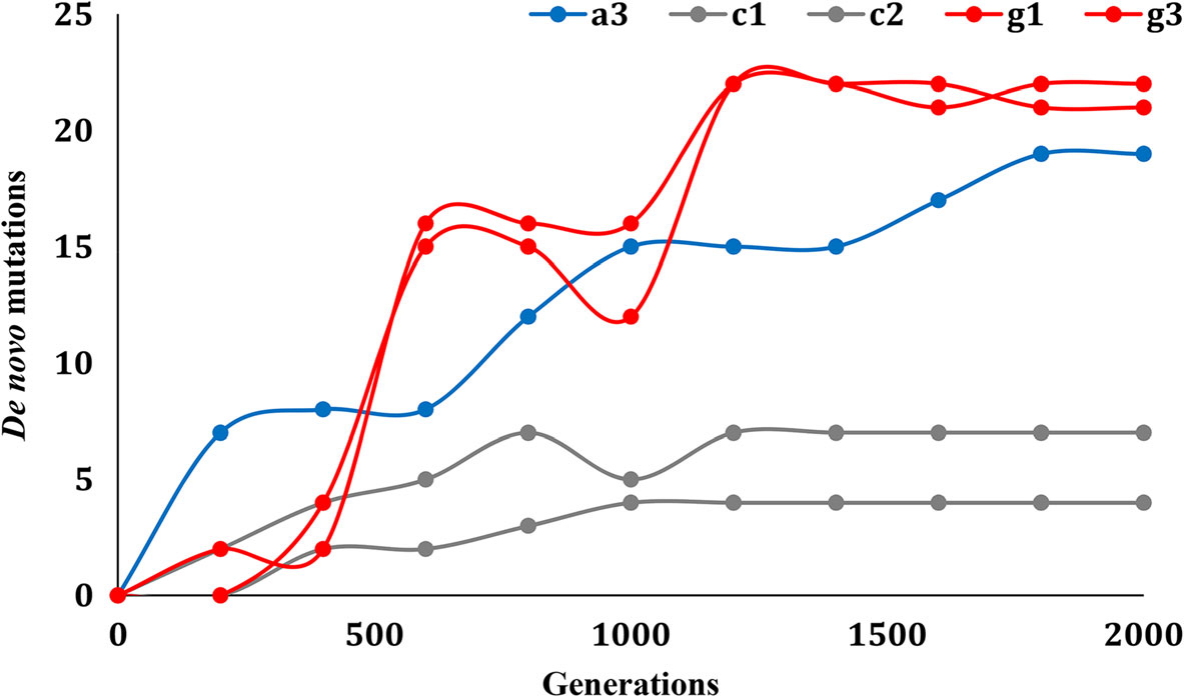
Mutation accumulation of *Lactobacillus casei* Zhang along the long-term cultivation in lactic acid bacteria susceptibility test medium (LSM) with and without antibiotics

The accumulation of *de novo* mutations along the long-term cultivation is presented in Fig. 1. We observed an around 4-fold increase in mutation accumulation (including SNVs, short insertions and deletions) in both strains A and G, comparing to the control (strain C) (i.e. an average of 19, 21.5, and 5.5 *de novo* mutations for strains A, G, and C, respectively). These corresponded to 0.01 *de novo* mutation per generation (3.5 × 10^-9^ per base pair per generation) under the amoxicillin or gentamicin selection pressure, comparing to 0.003 *de novo* mutation per generation (1.0 × 10^-9^ per base pair per generation) for the control. Such results are suggestive of apparent adaptive evolution of the *L. casei* Zhang genome, particularly when grown in the presence of antibiotics.

In parallel with the changes in gentamicin and amoxicillin MICs, the *de novo* mutation accumulation reached the maximum level at generation 1200 in strain G (Fig. 1). The mutation accumulation rate of strain A was highest during the first 400 generations (Fig. 1). Mutation accumulation still continued at a relatively high rate until generation 1000.

### Spectrum of *de novo* mutations

We further compared the spectrum of *de novo* mutations occurred in the three strains. We focused on the *de novo* SNVs because of the low number of observed small insertions and deletions (i.e. 1 insertion and 3 deletions in strain G, 1 deletion in the control, and no insertion or deletion in strain A), which would be inadequate for drawing any valid conclusions.

**Fig. 2:**
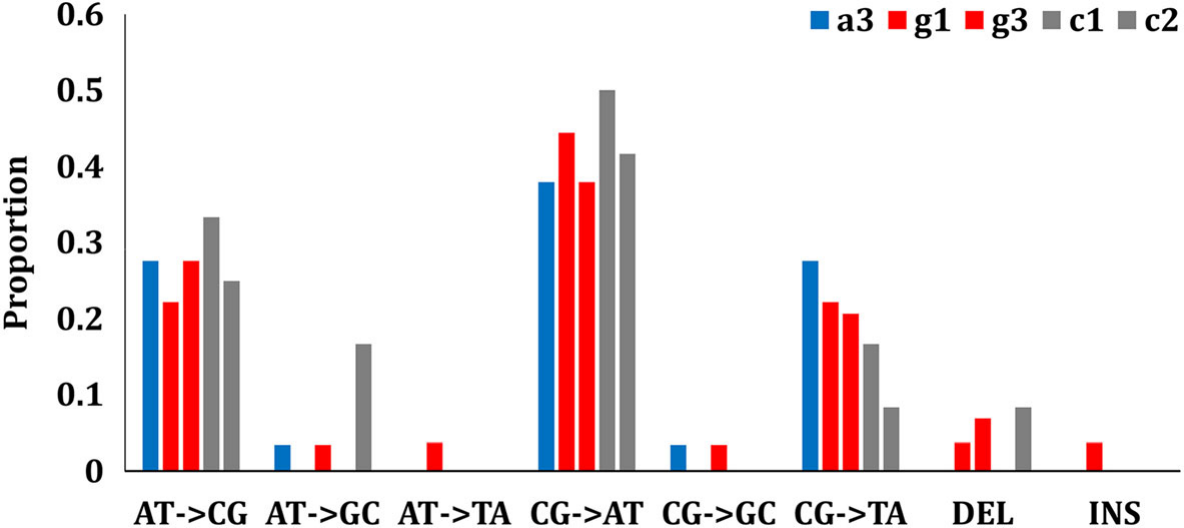
Spectrum of accumulated mutations of *Lactobacillus casei* Zhang along the long-term cultivation in lactic acid bacteria susceptibility test medium (LSM) with and without antibiotics

As illustrated in Fig. 2, AT- > CG and CG- > AT transversions were the two most common classes of *de novo* SNVs, contributing to 26.2% and 40.8% of the total detected mutations, respectively. Additionally, there was a high level of CG- > TA transition, i.e. 21.4% of the total detected mutations. No significant difference was observed between the mutation spectrum of either of the antibiotic exposed strains (strain A or G) and the control (strain C). This indicates that, as expected, the amoxicillin or gentamicin stress did not drive any specific type of mutations. The transition to transversion ratio (Ti/Tv) was 0.45, 0.33, and 0.29 for strains A, G, and C, correspondingly. Although the difference in Ti/Tv between strains A and C was 0.16, such difference could not be considered significant due to the limited number of identified mutations, especially in the control (strain C).

To demonstrate if positive selection had driven the evolution of strains A and G, we compared the fractions of non-synonymous mutations between strains with and without antibiotic treatment. We found significantly higher proportions of non-synonymous mutations in strains A (75%) and G (69%), comparing to the control (50%) (*P* = 0.029 and 0.049, respectively, determined by one-tailed z test for two proportions). The Ka/Ks ratio of strains A, G, and control were 1.02, 0.94, and 0.68, respectively. This shows that the control was protected from functional mutations, while the mutations in strains A and G occurred almost neutrally driven by amoxicillin and gentamicin application, correspondingly.

### Functional evaluation of the *de novo* mutations

**Fig. 3:**
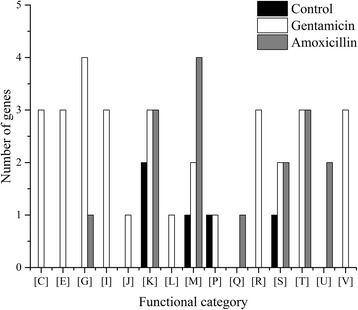
Clusters of orthologous groups (COG) of mutated genes of *Lactobacillus casei* Zhang during long-term cultivation in lactic acid bacteria susceptibility test medium (LSM) with and without antibiotics. Functional categories: [C], Energy production and conversion; [E], Amino acid transport and metabolism; [F], Nucleotide transport and metabolism; [G], Carbohydrate transport and metabolism; [H], Coenzyme transport and metabolism; [I], Lipid transport and metabolism; [J], Translation, ribosomal structure and biogenesis; [K], Transcription; [L], Replication, recombination and repair; [M], Cell wall/membrane/envelope biogenesis; [O], Posttranslational modification, protein turnover, chaperones; [P], Inorganic ion transport and metabolism; [Q], Secondary metabolites biosynthesis, transport and catabolism; [R], General function prediction only; [S], Function unknown; [T], Signal transduction mechanisms; [V], Defense mechanisms

Since non-synonymous mutations could possibly alter the functions of the coding genes, we assigned all the mutated genes into Clusters of Orthologous Groups (COGs) that classify genes based on functions. To estimate the number of mutated genes in different COG categories precisely, the same genes from the replicate cultures were considered as different genes and the number was accumulated. Common mutated genes that were shared across the amoxicillin, gentamicin, and control strains belonged mainly to two COG categories, transcription [K] and cell wall/membrane/envelope biogenesis [M] (Fig. 3). Compared to strains C and A, strain G had a more unique set of mutated genes, which was related to energy production and conversion [C], amino acid transport and metabolism [E], lipid transport and metabolism, translation [I], ribosomal structure and biogenesis [J], general function prediction only [R], and defense mechanisms [V].

Specifically, a Xenobiotic Response Element (XRE) family transcriptional regulator (LCAZH_1179) was found mutated in both strains A and G. The XRE family transcription factors are the second most frequently occurring regulator family among bacteria that participate in diverse metabolic functions [24]. Other putative genes that are involved in gene regulation included some transcriptional regulators (LCAZH_1767, LCAZH_1064, LCAZH_0070, and LCAZH_0059), some OmpR family DNA-binding response regulators (LCAZH_1669 and LCAZH_0490), a signal transduction histidine kinase (LCAZH_1668), a serine/threonine protein phosphatase (LCAZH_1613), a sensor histidine kinase *Prc*K (LCAZH_2350), and a DNA-directed RNA polymerase subunit beta (LCAZH_2481). Among them, the regulators of OmpR family is directly associated with antibiotic, extreme pH, temperature, and oxidative stresses [25]. Both of their coding proteins contain an N-terminal receiver (IPR001789) and a C-terminal DNA-binding domain (IPR001789).

Moreover, antibiotic-driven mutations were found in several other genes that are potentially associated with stress responses. Membrane modification is one of the key mechanisms of bacterial bile tolerance [26]. We have identified *de novo* mutations in seven genes that code for proteins relating to cell surface features, including a membrane carboxypeptidase (LCAZH_1472), a glycosyltransferase (LCAZH_2870), a mismatch repair ATPase (LCAZH_0590), a D-alanine-activating enzyme (LCAZH_0737), a cyclopropane fatty acid synthase-like methyltransferase (LCAZH_2067), and two FtsI/penicillin-binding proteins (LCAZH_1260 and LCAZH_1652). According to Denome et al. [27], three of these genes code for proteins (LCAZH_1472, LCAZH_1260, and LCAZH_1652) belonging to the penicillin-binding protein family that plays a role in the biosynthesis of cell wall peptidoglycan. Some members of this family act like glycosyltransferases, which are required for glycan chain polymerization [28]. Indeed, membrane modification has also been observed when bacteria are under acid stress. For example, when *L. casei* was adapting to an acidic environment, a cyclopropane fatty acid synthase-like methyltransferase was activated and expressed constitutively [29]. The enzyme catalyzes the addition of a methylene residue across the *cis* double bond of C_16:1n(9)_, C_18:1n(9)_, or C_18:1n(11)_ unsaturated fatty acids to form an unsaturated cyclopropane derivative. Another example is the D-alanine-activating enzyme, which was found to be associated with acid tolerance of *Streptococcus mutans* [30]. This enzyme catalyzes the first step of teichoic acids synthesis.

In response to external stresses, bacteria may modulate their energy metabolism. We located *de novo* mutations in the ATP synthase F0F1 subunits (LCAZH_1154 and LCAZH_1156), which are responsible for energy production and proton extrusion. Proton extrusion is also recognized as a mechanism of acid tolerance in bacteria [31].

## Discussion

Previously, Curragh and Collins reported a high spontaneous mutation frequency in *Lactobacillus* when the bacteria were cultivated with antibiotics, including nitrofurazone, kanamycin, and streptomycin; meanwhile, the bacterial antibiotic resistance increased together with the high spontaneous mutation frequency [13]. Similarly, we found an increased mutation frequency, i.e. ~4-fold higher, in strains grown in an amoxicillin- or gentamicin-containing environment, comparing to the control strain without antibiotic treatment, after subculturing for 2000 generations. Most of these mutations occurred before the first 1200 generations with a significantly higher proportion of non-synonymous mutations. Based on our results, the non-synonymous mutations were mostly found in genes involving in gene regulation and stress responses; thus, it is likely that they were function-driven, and they potentially contributed to the bacterial fitness adaptation. It is known that genetic changes may result in additional antibiotic resistance to pathogens that are not intrinsically present via various mechanisms, e.g. acquiring efflux system coding genes that pump out the antibiotic molecules, altering the antibiotics target enzymes, and degrading/modifying antibiotic molecules [32]. Whether the *de novo* mutations seen in our study confer additional resistance capacity to the strains cultivated with antibiotics remains to be determined in future studies.

The natural spontaneous base pair substitution (fixed) rate of *E. coli* was ~5-fold lower than that observed in *L. casei* Zhang (1.6 to 2.2 × 10^-10^ versus 1.0 × 10^-9^ per bp per generation for *E. coli* and *L. casei* Zhang, respectively) [2, 33]. However, under antibiotic stresses, the mutation frequency of *L. casei* Zhang was only one-third of that of *E. coli* (3.5 × 10^-9^ versus 1.1 × 10^-8^ per bp per generation) [2]. This suggests that although the genome of *L. casei* Zhang is less stable than that of *E. coli* under normal conditions, it may respond less to antibiotic stress. Further side-by-side comparative studies of different species are required to reach a more definite conclusion regarding whether high genome stability is a species-specific or universal characteristic for all probiotic bacteria.

A higher proportion of CG- > AT transversion was found in *L. casei* Zhang (40.8%; Fig. 2) compared to *E. coli* (23.5%) [2], even though the GC content is lower in the genome of *L. casei* Zhang (46.4% versus 50.6% in *E. coli*). The mutation spectrum of *L. casei* Zhang was similar, whether antibiotics were applied or not. Even without exposure to any external mutagen, *L. casei* Zhang exhibited a relatively high CG- > AT transversion rate. This may be a natural characteristic specific to this species due to a possible lack of the AG- or CT-mispair correction capacity [34].

Some pathogens have been shown to adapt rapidly upon antibiotic exposure by continuous accumulation of point mutations and thus raise serious health and safety concerns to the public [11]. However, unlike in the cases of pathogens, no significant increase in both mutation accumulation and MIC values was observed in *L. casei* Zhang in the later generations even with continuous antibiotic exposure. Instead, the mutation rate returned to the baseline level similar to that of the control, suggesting that both the bacterial genome and fitness level to antibiotics were stabilized. Our findings show that *L. casei* Zhang is a stable strain and presents limited biosafety risks even subject to long-term antibiotic exposure. However, the scope of the current study is limited to the analysis of mutation accumulation under in vitro cultivation conditions. Another important mechanism that drives evolution is horizontal gene transfer, which has not been addressed here. Particularly, when the probiotic bacteria arrive in the host gastrointestinal tract, in addition to antibiotic exposure, they will also be exposed to an enormous and complex microbial community within the colon. Horizontal gene transfer may then become a significant player in the genome stability and evolution of these bacteria in in vivo situations.

## Conclusions

In the present study, we monitored the genotypic and phenotypic changes of *L. casei* Zhang in a long-term evolution experiment with and without antibiotic (amoxicillin or gentamicin) selection pressure. We found that *L. casei* Zhang has high genome stability even subject to long-term antibiotic stresses. However, whether this is a species-specific or universal characteristic for all probiotic bacteria remains to be explored.

## Additional file

Additional file 1: Table S1. General information of the confirmed SNVs. (DOCX 23 kb)

## Abbreviations

ALE: Adaptive laboratory evolution
COGs: Clusters of orthologous groups
*E. coli*: *Escherichia coli*
*L. casei* Zhang: *Lactobacillus casei* Zhang
LAB: Lactic acid bacteria
LSM: LAB susceptibility test medium
MICs: Minimum inhibitory concentrations
MRS: de Mann-Rogosa-Sharpe
SNP: Single nucleotide polymorphism
SNV: Single nucleotide variation
