# Supplementary material for: Genome adaptive evolution of Lactobacillus casei under long-term antibiotic selection pressures
Source: BMC Genomics. 2017 Apr 24;18:320. doi: 10.1186/s12864-017-3710-x (PMC5402323; doi:10.1186/s12864-017-3710-x)
Supplement: Additional file 1: Table S1. — General information of the confirmed SNVs. (DOCX 23 kb) [file 12864_2017_3710_MOESM1_ESM.docx]

**Additional file 1: Table S1** General information of the confirmed SNVs

| Position | Reference | Mutation | Mutation type | Synonymous or nonsynonymous |
| --- | --- | --- | --- | --- |
| 856866 | G | T | SNV | Nonsynonymous |
| 1580780 | C | T | SNV | Nonsynonymous |
| 1613917 | G | A | SNV | Nonsynonymous |
| 1918488 | G | T | SNV | Nonsynonymous |
| 1955537 | G | A | SNV | Synonymous |
| 2141938 | C | T | SNV | Synonymous |
| 2315126 | A | C | SNV | Nonsynonymous |
| 2428926 | G | T | SNV | Nonsynonymous |
| 2459198 | C | A | SNV | Nonsynonymous |
